# The dual pathological roles and targeted therapy of PGE_2_: from receptor signaling networks to disease microenvironment modulation

**DOI:** 10.3389/fphar.2026.1810417

**Published:** 2026-07-15

**Authors:** Mingming Yang, Zihui Gao, Zihao Zhao, Xinxin Niu, Fei Xue, Jitai Zhang, Hualin Sun, Yuntian Shen, Guangliang Liu

**Affiliations:** 1 Department of Pediatrics, Binhai County People’s Hospital, Yancheng, Jiangsu, China; 2 Jiangsu Key Laboratory of Tissue Engineering and Neuroregeneration, Key Laboratory of Neuroregeneration of Ministry of Education, Co-Innovation Center of Neuroregeneration, Affiliated Hospital of Nantong University, Medical School of Nantong University, Nantong University, Nantong, Jiangsu, China

**Keywords:** EP receptors, mPGES-1, neuroinflammation, prostaglandin E2, targeted therapy, tumor microenvironment

## Abstract

Prostaglandin E_2_ (PGE_2_) is a pleiotropic lipid mediator that exerts context-dependent effects via four G protein-coupled receptors (EP1–EP4), playing a pivotal role in the pathogenesis of diverse disorders, including neurodegenerative, cardiovascular, neoplastic, and chronic inflammatory diseases. In this review, we systematically delineate the dualistic functions and mechanisms of PGE_2_ across these diseases. In neurodegenerative conditions such as Alzheimer’s and Parkinson’s diseases, PGE_2_ exacerbates neuroinflammation and neuronal injury in part through EP1 and EP2 in specific cell types, whereas EP4 signaling can confer neuroprotection in certain disease-stage and cellular contexts. Within the tumor microenvironment, PGE_2_ can drive immunosuppression, angiogenesis, and tumor progression via the EP2/EP4 axis, particularly in colorectal carcinoma, lung adenocarcinoma, and melanoma where this axis is best characterized. In cardiovascular and metabolic diseases, PGE_2_ exhibits both protective (EP4-mediated) and detrimental (EP3-mediated) effects. Building on this mechanistic framework, we highlight emerging therapeutic strategies designed to overcome the limitations of conventional non-steroidal anti-inflammatory drugs (NSAIDs). These include modulating key enzymes involved in PGE_2_ synthesis and degradation, developing subtype-selective EP receptor modulators for context-specific intervention, and synergistically targeting downstream pathogenic signaling pathways (e.g., PI3K/Akt/mTOR). By integrating mechanistic and translational perspectives, this review aims to advance next-generation therapies targeting the PGE_2_ signaling network.

## 1 Introduction

Arachidonic acid (AA), a key polyunsaturated fatty acid in membrane phospholipids, is released by phospholipase A_2_ (PLA_2_) and enzymatically converted into diverse eicosanoids, potent lipid mediators central to physiology and disease (Badimon et al., 2021). The active mediators are primarily generated through three classical metabolic pathways: the 5-lipoxygenase (5-LO) pathway producing leukotrienes (LTs), the cytochrome P450 pathway generating epoxyeicosatrienoic acids (EETs) and hydroxyeicosatetraenoic acids (HETEs), and the cyclooxygenase (COX) pathway, via COX-1 and COX-2, synthesizes prostaglandin H_2_ (PGH_2_), the precursor for thromboxane A_2_, prostacyclin, and notably, prostaglandin E_2_ (PGE_2_) (Kanaoka and Austen, 2019). PGE_2_, the most abundant prostaglandin in humans, acts locally and exhibits exceptionally diverse and context-dependent biological activities (Zhang et al., 2023).

PGE_2_ mediates a wide range of physiological and pathological processes, including regulation of vascular tone, smooth muscle contraction, inflammatory responses, and pain perception (Zhang et al., 2023). Its role is highly context-dependent and varies across diseases. PGE_2_ is a key driver in inflammatory diseases, promoting leukocyte activation and vascular permeability (Kawahara et al., 2015). In cancer, PGE_2_ shapes the tumor microenvironment to favor growth and metastasis (Wang and DuBois, 2018; Meng et al., 2025). In cardiovascular disease, PGE_2_ exhibits dual effects, with low doses promoting vasodilation and high doses potentially increasing cardiac burden (Malacarne et al., 2022). In neurodegenerative disorders such as Alzheimer’s and Parkinson’s disease, PGE_2_ signaling contributes to neuroinflammation and neuronal damage, yet also harbors receptor-specific neuroprotective potential (Johansson et al., 2015; Woodling et al., 2014). Given this complexity, understanding the precise mechanisms of PGE_2_ signaling offers critical insights for developing novel therapeutic strategies across multiple disease domains. This review summarizes the biosynthesis and receptor signaling of PGE_2_, with a focus on its complex roles in major diseases, aiming to provide new perspectives for future interventions.

A central conceptual tension runs through the biology reviewed here: the same lipid mediator activating the same receptor can produce diametrically opposite outcomes depending on cell type, disease chronicity, and signaling context. The characterization of EP receptor subtypes as “detrimental” or “protective” throughout this review should therefore be read as strictly context-dependent rather than absolute. Where evidence is mixed or mechanistically complex, we explicitly flag the key uncertainties and open questions, aiming to convey not only what is well-established but also why resolving the remaining ambiguities matters for therapeutic translation.

## 2 Metabolism and downstream receptors of PGE_2_

### 2.1 Metabolism of PGE_2_

**FIGURE 1:**
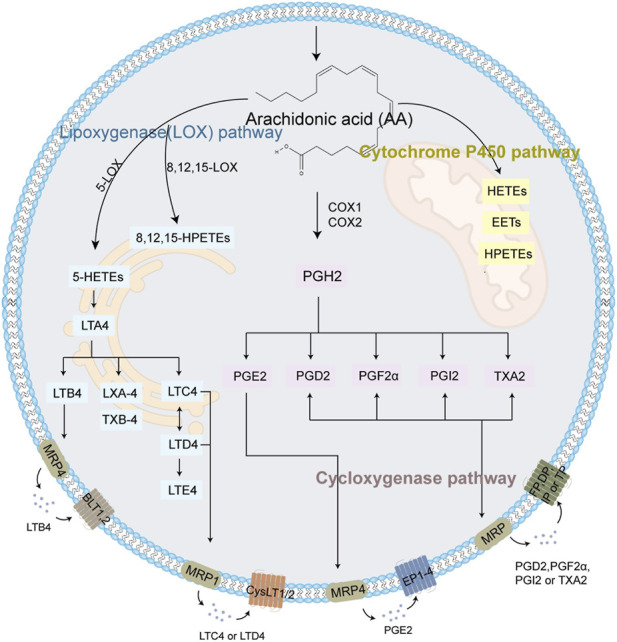
Three metabolic pathways of arachidonic acid. Arachidonic acid (AA) is metabolized via three classical enzymatic pathways. The COX-1/2 pathway converts AA to prostaglandin H_2_ (PGH_2_), which is further isomerized to PGE_2_, PGD_2_, PGI_2_, PGF_2_α, and thromboxane A_2_ (TXA_2_). These prostanoids signal through their cognate receptors (EP1–4, DP, IP, FP, TP), with PGE_2_ exported via MRP4. The 5-LOX pathway metabolizes AA to leukotriene A_4_ (LTA_4_) via 5-HPETE; LTA_4_ is hydrolyzed to LTB_4_ or conjugated to LTC_4_, which is further processed to LTD_4_. LTB_4_ signals through BLT receptors. The cytochrome P450 pathway oxidizes AA to epoxyeicosatrienoic acids (EETs), hydroxyeicosatetraenoic acids (HETEs), and hydroperoxyeicosatetraenoic acids (HPETEs).

The biosynthesis of PGE_2_ begins with the release of AA from membrane phospholipids, a step primarily catalyzed by PLA_2_ (Dennis and Norris, 2015). The balance among the COX, 5-LO, and CYP450 pathways determines the local eicosanoid profile and crucially influences whether PGE_2_-driven inflammation resolves or becomes chronic (Figure 1). Free AA is subsequently metabolized by COX, the rate-limiting enzyme of the prostaglandin synthesis pathway. The inducible isoform COX-2, which is significantly upregulated at sites of inflammation and disease, serves as a key driver for PGE_2_ production under pathological conditions (Seo and Oh, 2017). COX enzymes convert AA into the unstable intermediate prostaglandin H_2_ (PGH_2_), which is then transformed into various prostanoids, including PGE_2_, by specific terminal synthases.

The production of PGE_2_ is chiefly catalyzed by microsomal prostaglandin E synthase-1 (mPGES-1). This enzyme is functionally coupled with COX-2 during inflammatory responses and represents a critical regulatory node for PGE_2_ levels (Seo and Oh, 2017). Following its synthesis, PGE_2_ is actively secreted out of the cell via transporters such as multidrug resistance protein 4 (MRP4) (Pourmal et al., 2024), enabling it to act locally in an autocrine or paracrine manner. The spatial range of PGE_2_ signaling is tightly constrained by rapid extracellular degradation, predominantly mediated by the enzyme 15-hydroxyprostaglandin dehydrogenase (15-PGDH) (Zhang et al., 2015). This finely tuned regulation, from synthesis and export to inactivation, ensures the precise spatiotemporal control of PGE_2_ signaling.

The level of PGE_2_ in any pathological niche is not simply a function of COX-2 induction but reflects a dynamic equilibrium among substrate availability, competing metabolic pathways, transporter-mediated export, and enzymatic degradation. Arachidonic acid (AA) availability is regulated by phospholipase A_2_ (PLA_2_) isoform activity and membrane phospholipid composition; moreover, AA is simultaneously competed for by the 5-lipoxygenase (5-LO) and cytochrome P450 pathways. The balance between these routes determines the eicosanoid profile produced in response to a given stimulus and is a key determinant of whether inflammation resolves or becomes chronic—for example, defective lipid mediator class switching from prostaglandins to pro-resolving lipoxins observed in the tumor microenvironment (Section 3.3) and aging intestine (Section 3.4) reflects upstream dysregulation of this competitive flux. MRP4-mediated PGE_2_ secretion shapes the autocrine versus paracrine signaling radius through cell-type-specific expression differences between tumor cells and stromal cells. Finally, 15-PGDH functions not merely as a catabolic enzyme but as a dynamic rheostat of PGE_2_ bioavailability: its paradoxical upregulation in ALS astrocytes fails to suppress pathological PGE_2_ accumulation (Section 3.1), its age-dependent accumulation in skeletal muscle depletes regenerative PGE_2_ signaling (Section 3.4), and its pharmacological inhibition represents an emerging therapeutic strategy (Section 4.1).

### 2.2 Downstream receptors of PGE_2_

PGE_2_ elicits its diverse effects by activating four G protein-coupled receptor (GPCR) subtypes: EP1, EP2, EP3, and EP4 (Zhang et al., 2023). Each subtype exhibits a unique expression profile and G-protein coupling preference, underpinning the context-specific cellular responses to PGE_2_.

The EP1 receptor couples primarily to Gq, activating phospholipase C (PLC) to generate inositol trisphosphate (IP_3_) and diacylglycerol (DAG), thereby mobilizing intracellular calcium and stimulating protein kinase C (PKC) (Meng et al., 2025; Jiang et al., 2017). It mediates smooth muscle contraction, pain perception, and regulates cell survival. Its function is highly context-dependent, as it promotes apoptosis and neurotoxicity in the central nervous system—evident in models of Parkinson’s disease (Kawano et al., 2006)—while exhibiting anti-apoptotic effects in certain cancers (Niu et al., 2019). The EP2 and EP4 receptors both primarily signal through Gs, stimulating adenylyl cyclase (AC) to elevate intracellular cAMP and activate protein kinase A (PKA) (Zhang et al., 2018; Yokoyama et al., 2013; Qu et al., 2021). Despite this common pathway, EP4 demonstrates greater signaling versatility, additionally coupling to Gi, PI^3^K, β-arrestin, and β-catenin, which enables it to mediate both anti-inflammatory and pro-inflammatory responses (Yokoyama et al., 2013; Huang et al., 2023). EP2 is highly expressed in the uterus, lungs, and spleen, and is involved in processes such as tracheal dilation and vascular smooth muscle relaxation. Its role in driving neuroinflammation is well-documented in models of Alzheimer’s disease (AD) and amyotrophic lateral sclerosis (ALS) (Johansson et al., 2015; Kosuge et al., 2018). The EP3 receptor is unique for its primary coupling to Gi, leading to inhibition of AC and a reduction in cAMP levels (Suno et al., 2022). Generated by alternative splicing, its multiple isoforms may exhibit differential signaling. EP3 is critically involved in inflammation and immune regulation (Natura et al., 2013). In the cardiovascular system, EP3 activation can suppress cardiac contractility and exacerbate post-infarction injury (Maxwell et al., 2023), while in the kidney, it helps regulate water and sodium balance (Li et al., 2018). Thus, the combinatorial diversity in G-protein coupling, effector recruitment, and spatiotemporal expression of the EP receptors underpins the pleiotropic roles of PGE_2_, presenting precise targets for therapeutic intervention.

Three additional mechanistic dimensions fundamentally shape the context-dependence of PGE_2_ effects. First, EP2 and EP4 differ critically in signaling kinetics: EP4 undergoes rapid β-arrestin-mediated desensitization and internalization, producing transient cAMP accumulation that may favor pro-resolution signaling, whereas EP2 is largely resistant to desensitization and sustains cAMP elevation over prolonged periods. In chronic disease states with persistently elevated PGE_2_, this kinetic asymmetry may mean that EP2-driven sustained signaling disproportionately contributes to maladaptive outcomes (Yokoyama et al., 2013; Qu et al., 2021). Second, EP4 exhibits pronounced pathway-biased signaling: depending on cellular context, EP4 can preferentially engage Gs/cAMP/PKA (anti-inflammatory and cardioprotective), Gi (attenuating cAMP), PI3K/Akt (pro-survival and implicated in tumorigenesis), or β-arrestin (scaffolding G-protein-independent complexes). This bias may explain why EP4 agonism is protective in the ischemic heart but pro-tumorigenic in the tumor microenvironment, and raises the possibility that biased EP4 ligands selectively engaging Gs/cAMP while avoiding PI3K/β-arrestin could offer superior therapeutic selectivity (Yokoyama et al., 2013; Huang et al., 2023). Third, EP receptors can signal from internalized endosomal compartments with distinct biological consequences, and cAMP is organized into spatially discrete nanodomains regulated by phosphodiesterases and AKAPs—a dimension of spatial compartmentalization that is likely to contribute to the context-dependent effects of PGE_2_ signaling across tissues and disease states (Vleeshouwers et al., 2020; Yadav and Zaccolo, 2025; Brighton et al., 2024). Together, signaling kinetics, pathway-biased signal transduction, and spatial compartmentalization constitute a mechanistic framework that reconciles the context-dependent pleiotropy of PGE_2_-EP signaling across tissues and disease states. It should be noted, however, that clinically validated biased EP4 ligands do not yet exist; this concept remains at the preclinical and theoretical stage, and its translational feasibility awaits pharmacological proof-of-concept in disease models.

## 3 PGE_2_ involving physiological progress and diseases

### 3.1 The role of PGE_2_ in neurodegenerative diseases

**FIGURE 2:**
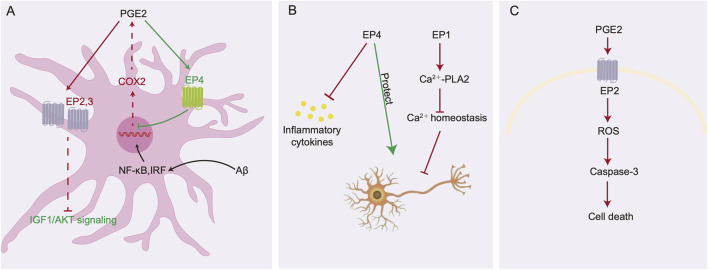
PGE_2_ signaling pathways in neurodegenerative diseases. **(A)** Alzheimer’s disease (AD): Aβ oligomers activate microglial IRF/NF-κB pathways, upregulating COX-2/PGE_2_/EP2 signaling and suppressing IGF1/AKT. EP4 signaling counteracts neuroinflammation. **(B)** Parkinson’s disease (PD): EP1 activation exacerbates Ca^2+^-dependent neurotoxicity, whereas EP4 activation confers neuroprotection. **(C)** Amyotrophic lateral sclerosis (ALS): PGE_2_ engages EP2 on NSC-34 motor neuron-like cells, inducing oxidative stress and caspase-3-dependent apoptosis.

Neuroinflammation is a critical driver of neurodegenerative diseases. PGE_2_, a key inflammatory mediator, exhibits complex and often opposing effects within the central nervous system, dictated by its signaling through specific EP receptors. This section explores the dual roles of PGE_2_ in Alzheimer’s disease (AD), Parkinson’s disease (PD), and amyotrophic lateral sclerosis (ALS), highlighting how selective receptor targeting presents promising therapeutic strategies. Despite distinct upstream pathologies, the cell-type-specific receptor dominance patterns across AD, PD, and ALS converge on a common therapeutic logic: suppressing EP2/EP1 while activating EP4 (Figure 2).

Alzheimer’s Disease (AD) is characterized by amyloid-beta (Aβ) plaque deposition, tau pathology, and chronic neuroinflammation. PGE_2_ levels in cerebrospinal fluid are dynamically altered during AD progression and are considered a potential biomarker (Forte et al., 2024). Beyond Aβ, factors like sleep disorders may exacerbate AD pathology through shared neuroinflammatory pathways, further implicating the PGE_2_ system (Zhang et al., 2025a). In pathogenesis, Aβ oligomers activate inflammatory pathways linked to upregulated COX/PGE_2_/EP2 signaling. Recent research directly confirms that targeting the COX-1/PGE_2_/EP2 signaling axis effectively alleviates microglia-induced neuroinflammation and mitigates cognitive impairment in AD models (Wang J. et al., 2025). Specifically, PGE_2_ signaling through the EP2 receptor on aged myeloid cells disrupts cellular metabolism, driving a pro-inflammatory state; blocking this signaling can restore cognitive function (Minhas et al., 2021). Conversely, EP4 signaling exerts anti-inflammatory effects, suppressing neuroinflammation and enhancing microglial phagocytic function (Woodling et al., 2014). Thus, a therapeutic strategy emerges: suppressing detrimental EP2 signaling while preserving or augmenting neuroprotective EP4 activity.

Parkinson’s Disease (PD) involves the selective loss of dopaminergic neurons, with neuroinflammation and α-synuclein aggregation jointly driving disease progression (Yildirim-Balatan et al., 2024). PGE_2_ synthesis is upregulated in PD, and its inhibition alleviates dopaminergic neuron loss (Ikeda-Matsuo et al., 2019). PGE_2_ signaling exhibits clear receptor specificity: EP1 activation enhances neurotoxicity via Ca^2+^-dependent pathways in both patients and models (Kawano et al., 2006), whereas EP4 signaling demonstrates potent neuroprotective and anti-inflammatory properties in acute dopaminergic injury (Pradhan et al., 2017). Therefore, targeting specific EP receptors—particularly inhibiting EP1 while preserving EP4 signaling—represents a promising strategy for modulating neuroinflammation in PD.

Amyotrophic Lateral Sclerosis (ALS) is characterized by the degeneration of motor neurons (Shen et al., 2024; Sun et al., 2022; Yang et al., 2021). Elevated spinal cord PGE_2_ levels are found in patients and models. Genetic or pharmacological blockade of the EP2 receptor dampens neuroinflammation, improves motor function, and extends survival, highlighting its central role (Kosuge et al., 2018; Nango et al., 2023). *In vitro*, PGE_2_ activates EP2 on motor neuron-like cells, inducing caspase-3-dependent apoptosis via oxidative stress. Interestingly, despite upregulation of the PGE_2_-degrading enzyme 15-PGDH in astrocytes, PGE_2_ levels remain high, suggesting impaired catabolism (Miyagishi et al., 2017). Targeting the dysregulated PGE_2_-EP2 axis, particularly in glial cells, is thus a compelling therapeutic avenue for ALS.

In summary, across AD, PD, and ALS, PGE_2_ signaling orchestrates critical neuroinflammatory processes. The consistent theme is the detrimental role of the EP2 receptor in driving disease progression, while the EP4 receptor often confers protection. This precise dichotomy, as illustrated in Figure 2, provides a clear rationale for developing receptor-subtype-selective therapies to intervene in these currently incurable diseases. It is important to note, however, that this EP2/EP4 functional dichotomy is strictly context- and cell-type-dependent. The detrimental role of EP2 is best supported in aged or chronically activated myeloid cells during sustained neuroinflammation; early-stage EP2 signaling may initially serve adaptive functions such as promoting phagocytic clearance, and the consequences of EP2 signaling differ across microglia, astrocytes, and neurons. Key open questions remain: What determines EP receptor subtype dominance in a given pathological niche? How does disease chronicity alter the balance between EP2-sustained and EP4-transient cAMP signaling? Can biased EP4 ligands be designed to selectively activate neuroprotective while avoiding oncogenic pathways? This last question, while conceptually compelling, must be tempered by the recognition that no clinically validated biased EP4 ligands currently exist; the strategy remains at the preclinical and theoretical stage, and its translational feasibility awaits pharmacological proof-of-concept. Resolving these uncertainties is essential for translating receptor-selective therapeutic logic into safe and effective clinical strategies.

### 3.2 The role of PGE_2_ in cardiovascular disease

**FIGURE 3:**
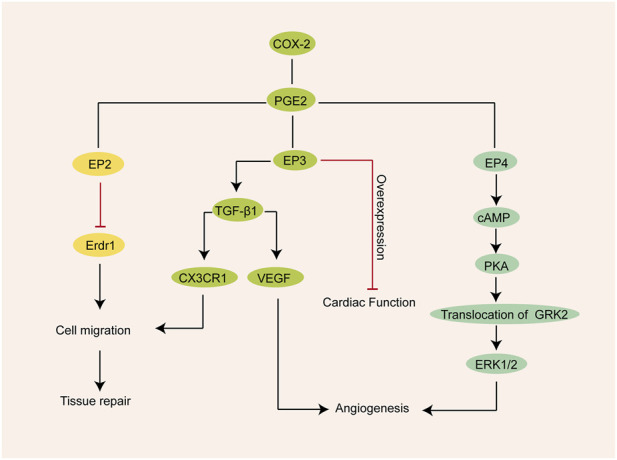
PGE_2_ signaling in the cardiovascular system. PGE_2_ acts through EP2, EP3, and EP4 receptors to mediate divergent cardiac and vascular outcomes. EP2 signaling suppresses Erdr1 and promotes macrophage migration, facilitating cardiac repair. EP3 activation enhances macrophage CX3CR1 expression via TGF-β1 and stimulates VEGF-dependent angiogenesis; however, cardiomyocyte-specific EP3 overexpression exacerbates post-infarction dysfunction. EP4 signaling drives GRK2 translocation to the plasma membrane via cAMP/PKA, reducing GRK2–ERK1/2 interaction and promoting ERK1/2-mediated angiogenesis.

PGE_2_ exerts complex, receptor-specific effects on cardiovascular homeostasis and pathology, balancing protective healing with potential maladaptive remodeling. The divergent outcomes of EP2, EP3, and EP4 activation in the cardiovascular system highlight a key cell-type-dependent duality—for example, EP3 is beneficial in macrophages but detrimental in cardiomyocytes (Figure 3). Its synthesis shifts under stress, with inducible COX-2 driving altered PGE_2_ levels that critically influence disease outcomes (Ricciotti et al., 2024). Recent insights extend beyond PGE_2_ itself to encompass the balance of its lipid precursor network; for instance, elevated levels of AA are associated with cardioprotection and can synergize with low-dose aspirin to enhance efficacy against ischemia-reperfusion injury while potentially mitigating bleeding risk (Talabieke et al., 2025).

PGE_2_ primarily signals through EP2, EP3, and EP4 receptors to modulate inflammation, repair, and cardiac remodeling. A key mechanism is the regulation of macrophage function. PGE_2_ drives macrophage polarization toward an anti-inflammatory, reparative M2 phenotype, facilitating tissue healing. This is mediated in part by EP2, as its deficiency impairs M2 recruitment and cardiac repair (Wu et al., 2018), a pathway that underlies the therapeutic benefit of interventions such as stem cell therapy in heart injury (Zhang et al., 2025b). The PGE_2_-EP3 axis also contributes to healing by enhancing macrophage migration and angiogenesis (Tang et al., 2017). However, EP3 signaling exhibits a critical cell-type-dependent duality; while beneficial in immune cells, its specific overexpression in cardiomyocytes exacerbates dysfunction and adverse remodeling after injury (Maxwell et al., 2023).

In contrast, EP4 receptor activation is predominantly cardioprotective. It attenuates myocardial ischemia-reperfusion injury, reduces infarct size, and improves function by suppressing detrimental inflammation and remodeling (Bryson et al., 2018; Kanno et al., 2023). EP4 also promotes therapeutic angiogenesis, a process involving cAMP/PKA signaling as depicted (Han et al., 2020). Notably, the protective role of EP4 extends to the cerebrovascular system, where it mediates critical vasodilation and increased blood flow following cerebral ischemia, highlighting its broader significance in vascular health (Chen et al., 2025). In atherosclerosis, although EP4 deficiency may not alter plaque size, it exacerbates intra-plaque inflammation, underscoring its role in promoting plaque stability (Tang et al., 2011).

Thus, PGE_2_’s impact is dichotomous and context-defined: detrimental signaling often via cardiomyocyte EP3 contrasts with the protective, reparative functions mediated by EP2 on macrophages and EP4 broadly. This receptor-specific duality, coupled with insights into precursor metabolism (e.g., AA), unveils precise therapeutic targets for myocardial infarction, heart failure, and atherosclerosis. Notably, the protective role of EP4 signaling extends to the cerebrovascular system, highlighting the broader therapeutic potential of modulating the PGE_2_ pathway across cardiovascular and cerebrovascular disorders. Importantly, EP4 activation is not universally protective in the vasculature: EP4-mediated PI3K/Akt signaling has been associated with vascular smooth muscle cell proliferation and neointimal hyperplasia following vascular injury—a context where pro-proliferative activity may be maladaptive (Xu et al., 2022). This parallels EP4’s pro-tumorigenic signaling in the TME (Section 3.3), illustrating that the same receptor can mediate beneficial or harmful outcomes depending on cell identity and injury context. EP3 signaling demonstrates the same cell-type dependence: beneficial in immune cells yet exacerbating dysfunction when overexpressed in cardiomyocytes—underscoring that conclusions about receptor function cannot be extrapolated across cell types even within the same organ.

### 3.3 The role of PGE_2_ in tumors (cancer)

**FIGURE 4:**
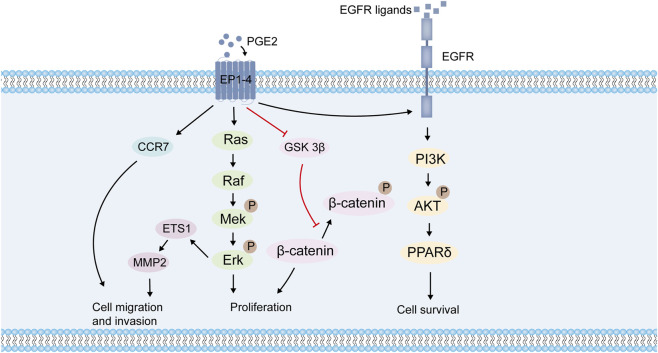
PGE_2_ regulates multiple oncogenic signaling pathways. PGE_2_ promotes cancer cell proliferation, survival, migration, and invasion through EP receptor-dependent cascades. For proliferation, EP2 engagement inhibits GSK3β, stabilizing β-catenin and driving nuclear translocation and target gene transcription. For survival, EP4 activates the PI3K/Akt/PPARδ pathway. For migration and invasion, PGE_2_ transactivates EGFR, leading to PI3K/AKT activation; additionally, it upregulates the Erk–ETS1–MMP2 axis and CCR7 expression.

A note on scope: the mechanisms described in this section are best supported by evidence from colorectal carcinoma, lung adenocarcinoma, and melanoma—tumor types with the most extensive literature on PGE_2_-EP signaling. The extent to which these mechanisms apply broadly across cancer histologies remains an active area of investigation, and conclusions should be interpreted as context-specific rather than pan-cancer. PGE_2_ signaling and EP receptor usage differ substantially by tumor type, disease stage, and immune infiltrate composition. PGE_2_ plays a central role in tumor progression through mechanisms that encompass both the direct promotion of cancer cell malignancy and the orchestration of an immunosuppressive tumor microenvironment (TME). Epidemiological evidence links the chemopreventive effects of non-steroidal anti-inflammatory drugs (NSAIDs) to the inhibition of the COX-2/PGE_2_ axis (Zaman et al., 2022). The mechanisms by which PGE_2_ promotes tumor cell proliferation, survival, migration, and invasion show that multiple oncogenic cascades—β-catenin/Wnt, PI3K/Akt, EGFR transactivation, MAPK/ERK, and CCR7/MMP2 — are simultaneously activated via EP2 and EP4, meaning that blocking the upstream EP receptors can suppress several cancer hallmarks at once (Figure 4). Mechanistically, tumor-derived PGE_2_ acts in an autocrine and paracrine manner, primarily via the EP2 and EP4 receptors. It transactivates the epidermal growth factor receptor (EGFR), initiating the PI3K/AKT survival cascade (Pai et al., 2002; Finetti et al., 2023). A pivotal mechanism involves PGE_2_ binding to EP2, which triggers a unique Gs protein-mediated disruption of the β-catenin degradation complex. This leads to β-catenin stabilization, nuclear translocation, and the transcriptional activation of pro-proliferative genes, a hallmark in cancers like colorectal carcinoma (Castellone et al., 2005; Castellone et al., 2006). Furthermore, PGE_2_ activates the Ras/MAPK pathway to stimulate proliferation and enhances cell migration and invasion through the upregulation of effectors such as matrix metalloproteinase-2 (MMP2) and the chemokine receptor CCR7 (Chen et al., 2024; Pan et al., 2008; Wang et al., 2022).

**FIGURE 5:**
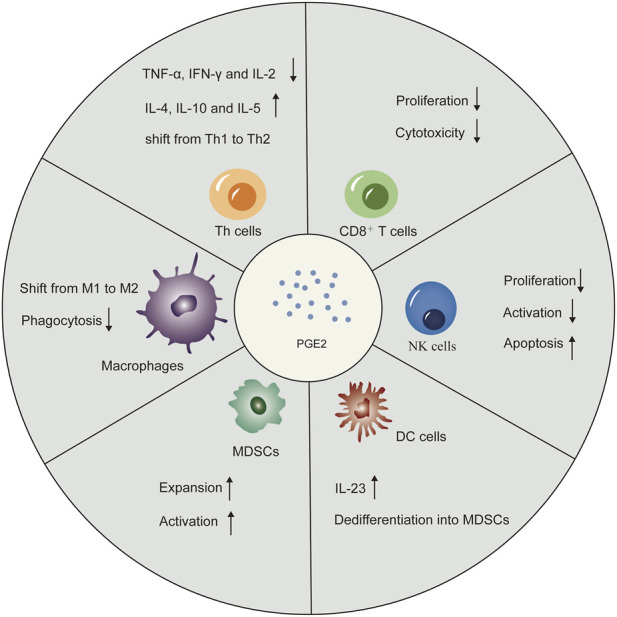
PGE_2_ reprograms the tumor immune microenvironment. PGE_2_ suppresses anti-tumor immunity through multiple mechanisms. It inhibits Th1 cytokines (TNF-α, IFN-γ, IL-2) and promotes Th2 cytokines (IL-4, IL-10, IL-5), shifting the Th1/Th2 balance. PGE_2_ drives macrophage M2 polarization, impairing phagocytosis. It upregulates IL-23 in dendritic cells (DCs) and induces DC dedifferentiation into myeloid-derived suppressor cells (MDSCs). PGE_2_ also suppresses natural killer (NK) cell activation and proliferation and promotes NK cell apoptosis.

Concurrently, PGE_2_ is a master regulator of the TME, subverting anti-tumor immunity to foster a permissive niche for growth and metastasis. The multiple immunosuppressive mechanisms orchestrated by PGE_2_-EP2/EP4 signaling—including Th1/Th2 shift, Treg programming, MDSC expansion, M2 polarization, and NK cell inhibition—form a multi-pronged barrier that is difficult to appreciate when each mechanism is described separately (Figure 5). It drives a critical shift in CD4^+^ T helper cell polarization, suppressing pro-inflammatory Th1 cytokines (e.g., TNF-α, IFN-γ, IL-2) while promoting anti-inflammatory Th2 cytokines (e.g., IL-4, IL-5, and IL-10), thereby impairing effective cellular immunity (Wang et al., 2022; Stolina et al., 2000). This pro-tumorigenic state is further cemented by a failure to resolve inflammation and the active shaping of immunosuppressive cells. Specifically, defective “lipid mediator class switching” sustains a chronic pro-inflammatory TME, while PGE_2_ signaling via EP2/EP4 receptors directly programs tumor-infiltrating regulatory T cells (Tregs) for enhanced suppressive function (Matsuura et al., 2025; Soundararajan et al., 2025). PGE_2_ potently expands and activates myeloid-derived suppressor cells (MDSCs) and skews tumor-associated macrophages (TAMs) toward a pro-tumorigenic M2 phenotype (Yan et al., 2018; Porta et al., 2020). These immunosuppressive myeloid populations, in turn, inhibit the cytotoxic function of CD8^+^ T cells and natural killer (NK) cells (Mizuno et al., 2019). A specific mechanism underlying NK cell inhibition involves tumor-derived PGE_2_ activating the immunosuppressive cAMP/CREM signaling pathway within NK cells (Rothfuß et al., 2025). This multifaceted reprogramming of the immune landscape establishes a robust barrier against anti-tumor responses, highlighting the EP2/EP4 signaling axis as a compelling therapeutic target for reversing immune evasion. Multiple distinct dysfunctions—Treg expansion, MDSC recruitment, NK cell inhibition, and CD8^+^ T cell exhaustion—thus converge on the EP2/EP4 axis. This convergence underpins the concept of immune circuit reprogramming as the central therapeutic objective: EP receptor blockade may, in principle, reverse multiple arms of the PGE_2_-driven immunosuppressive network simultaneously—though the extent to which this holds across tumor types and stages remains to be established—rather than targeting a single immunosuppressive mechanism in isolation.

### 3.4 The role of PGE_2_ in aging

The role of PGE_2_ in aging is complex and highly tissue-specific, exhibiting dual and often opposing effects on immune function and tissue regeneration. Emerging evidence underscores that dysregulation of eicosanoid biosynthesis, including PGE_2_ signaling, is a hallmark of immunosenescence and tissue aging across multiple organs (Burkett et al., 2023).

In the context of immunosenescence and chronic inflammation, elevated PGE_2_ significantly impairs adaptive immunity by suppressing CD4^+^ T cell proliferation and IL-2 production (Burkett et al., 2023). This dysregulated signaling extends to other eicosanoid pathways and organ systems. For instance, in the aged lung, type II alveolar epithelial cells secrete excessive PGE_2_ in response to influenza A virus infection, which via the EP2 receptor limits alveolar macrophage proliferation and compromises their mitochondrial function, thereby weakening pulmonary defense (Chen et al., 2022). Furthermore, aging disrupts the resolution of intestinal inflammation through a defective “eicosanoid-immune-microbiota axis,” where impaired lipid mediator class switching and sustained PGE_2_ signaling through receptors like EP4 contribute to persistent, low-grade gut inflammation and microbiome dysbiosis, a key feature of aging gut (Goepp et al., 2025). Paradoxically, in stark contrast to its immune-inhibitory role, PGE_2_ is crucial for maintaining tissue integrity, stem cell function, and regenerative capacity in other aging organs. In skeletal muscle, aging is associated with a significant reduction in local PGE_2_ levels, concomitant with the accumulation of its degrading enzyme, 15-hydroxyprostaglandin dehydrogenase (15-PGDH). Multi-omics studies reveal that aged muscle stem cells exhibit blunted PGE_2_-EP4 receptor signaling, leading to precocious differentiation commitment and mitotic catastrophe, which underpins regenerative failure. Restoring PGE_2_ signaling rejuvenates these stem cells, reversing muscle atrophy and restoring strength (Palla et al., 2021; Wang Y. X. et al., 2025). Beyond autonomous muscle repair, PGE_2_ exerts systemic neurotrophic effects. In models of sciatic nerve injury in aged rats, intramuscular delivery of PGE_2_ activates the cAMP response element binding protein (CREB) in spinal motor neurons, stimulating axonal regeneration (Bakooshli et al., 2023).

Thus, aging involves a profound, tissue-contextual imbalance in PGE_2_ signaling. It becomes detrimentally overactive in driving immune suppression and unresolved inflammation in organs like the lung and gut, yet becomes deficient in supporting stem cell function and regeneration in tissues like muscle. This duality underscores a promising therapeutic strategy: context-dependent modulation of the PGE_2_ pathway—aimed at suppressing its immuno-inhibitory actions (e.g., via EP2/EP4 antagonism at sites of chronic inflammation) while restoring its anabolic and regenerative functions (e.g., via 15-PGDH inhibition or local PGE_2_/EP4 agonism in muscle and nerve)—to counteract multiple facets of age-related decline.

### 3.5 The role of PGE_2_ in chronic inflammatory and autoimmune diseases

PGE_2_ is a central lipid mediator that orchestrates diverse pathophysiological processes across chronic inflammatory and autoimmune diseases through its spatially and temporally regulated signaling via four distinct E-type prostanoid (EP) receptors. Its actions are highly context-dependent, influencing metabolism, tissue remodeling, and immune cell fate, thereby presenting both challenges and opportunities for therapeutic intervention.

In metabolic disorders such as obesity and type 2 diabetes, PGE_2_ derived from adipose tissue macrophages plays a nuanced role. Recent evidence indicates that macrophage-specific EP3 signaling protects against diet-induced obesity by upregulating the matricellular protein SPARC, which is involved in tissue remodeling and metabolic regulation. Disruption of this pathway exacerbates adiposity and inflammation, revealing a non-canonical protective role for PGE_2_-EP3 signaling in metabolic homeostasis (Shang et al., 2025). Conversely, in pancreatic islets, signaling through the EP4 receptor has been shown to promote β-cell survival and proliferation, highlighting the receptor-specific duality of PGE_2_ action in metabolic tissues (Carboneau et al., 2017). Within the skeletal system, PGE_2_ is a key driver of osteoarthritis (OA) pathology. It is hypersecreted by subchondral bone osteoblasts and acts predominantly via the EP4 receptor on osteoclasts to promote pathological bone remodeling and pain (Jiang et al., 2022). This receptor-specific action makes the EP4 axis a compelling target for OA treatment, potentially offering analgesia and disease modification beyond the scope of general COX-2 inhibition.

The role of PGE_2_ in autoimmune pathologies is multifaceted, particularly in shaping adaptive immune responses. In rheumatoid arthritis (RA), PGE_2_ signaling promotes the differentiation and expansion of pathogenic Th1 and Th17 cells. Emerging research has begun to delineate the upstream regulators of this pathway. For instance, endoplasmic reticulum-mitochondria coupling stress in mesenchymal stem cells can enhance their therapeutic efficacy for RA by activating the eIF2α-ATF4 pathway, which in turn upregulates COX-2/PGE_2_ production to suppress pro-inflammatory follicular helper T cells (Liu et al., 2025). Furthermore, macrophage lipid metabolism is intricately linked to PGE_2_ biosynthesis; modulation of PPARγ-mediated lipid accumulation can effectively suppress PGE_2_ release and M1 macrophage polarization, presenting another viable intervention node (Jiang et al., 2026). In central nervous system autoimmunity, such as multiple sclerosis (MS), advanced spatial mapping technologies have provided unprecedented insight. Spatial metabolomics of MS lesions reveals a dysregulated arachidonic acid cascade with a distinct topography of PGE_2_ and its EP receptors within active lesion areas, directly linking localized PGE_2_ biosynthesis to the chronic inflammatory microenvironment (Hansen et al., 2025). This spatial precision underscores the complexity of targeting this pathway in compartmentalized diseases. Beyond these focal points, PGE_2_ receptors are spatially distributed throughout the kidney to finely regulate hemodynamics and sodium handling, playing a critical role in maintaining renal function and contributing to the pathogenesis of various kidney diseases (Li et al., 2018). In summary, PGE_2_ functions as a master regulator in chronic inflammation and autoimmunity, with effects spanning from metabolic control and tissue damage to precise immune cell instruction. The evolving understanding of its upstream regulatory mechanisms—including cellular stress responses, metabolic reprogramming, and spatial localization—points toward a new generation of therapeutic strategies. Moving beyond broad receptor antagonism, future approaches may involve precision targeting of specific biosynthetic nodes or receptor subtypes in defined cellular and anatomical contexts to uncouple detrimental inflammation from homeostatic or protective signals.

## 4 Therapeutic targeting and strategies for the PGE_2_ signaling pathway

PGE_2_ is a master regulator of immunometabolic homeostasis, and its dysregulation underpins a wide range of diseases, from neurodegeneration and cancer to metabolic and autoimmune disorders. Moving beyond non-selective COX inhibition, contemporary strategies focus on the precision modulation of the PGE_2_ axis. This includes targeting its biosynthetic and catabolic enzymes, selectively engaging its four EP receptors (EP1-EP4), and intervening in key downstream signaling cascades. These approaches aim to overcome the limitations of conventional NSAIDs by enabling tissue-specific, context-dependent interventions with superior safety and efficacy profiles.

### 4.1 Targeting PGE_2_ synthesis and degradation pathways

Conventional NSAIDs inhibit COXs to reduce PGE_2_ but compromise the synthesis of protective prostaglandins (e.g., PGI_2_), leading to gastrointestinal and cardiovascular side effects. A refined strategy involves selectively inhibiting microsomal prostaglandin E synthase-1 (mPGES-1), the inducible terminal enzyme for pathological PGE_2_ overproduction, while sparing constitutive pathways (Rahman et al., 2025). Conversely, enhancing PGE_2_ degradation presents a complementary approach. Prostaglandin degradation is primarily mediated by 15-hydroxyprostaglandin dehydrogenase (15-PGDH). In ageing muscle, 15-PGDH overexpression depletes PGE_2_ and contributes to atrophy, whereas its pharmacological inhibition or exogenous PGE_2_ supplementation rescues muscle mass and function (Palla et al., 2021; Bakooshli et al., 2023). Thus, a dual strategy combining upstream suppression of mPGES-1 with downstream modulation of 15-PGDH activity offers a synergistic and potentially safer avenue for treating conditions of pathological PGE_2_ accumulation.

To aid translational evaluation, the therapeutic strategies discussed in this section can be stratified by stage of development. Tier 1 (most clinically advanced): COX-2 inhibitors and NSAIDs provide extensive human safety and efficacy data but lack EP subtype selectivity and carry well-characterized cardiovascular and gastrointestinal liabilities; mPGES-1 inhibitors have entered Phase I/II trials with improved selectivity profiles relative to COX inhibitors. Tier 2 (active clinical investigation): EP4 antagonists in oncology (e.g., E7046/ONA-255) have demonstrated preliminary immune activation signals in Phase I/II settings. Tier 3 (preclinical/emerging): 15-PGDH inhibitors for tissue regeneration, biased EP4 agonists, and compartment-targeted delivery systems remain at proof-of-concept or early preclinical stages. EP3 antagonists have shown strong preclinical validation in metabolic disease models, but have not yet entered clinical trials for these indications (Shang et al., 2025; Chan et al., 2016). Three cross-cutting challenges apply across all tiers (Badimon et al., 2021): receptor cross-talk—antagonizing one EP subtype may increase ligand availability for others, potentially undermining efficacy or generating off-target effects (Kanaoka and Austen, 2019); tissue-specific delivery—achieving therapeutic concentrations in the CNS or tumor core without systemic immunosuppression or cardiovascular liability remains formidable (Zhang et al., 2023); context-dependent safety—EP4 agonism that is cardioprotective may be pro-tumorigenic in patients with occult malignancy, necessitating careful patient stratification and biomarker development.

### 4.2 Targeting downstream EP receptors for disease-specific strategies

The structural elucidation of EP receptors provides a blueprint for rational drug design. Recent structural studies reveal subtype-specific features that dictate G-protein coupling selectivity (e.g., EP4/EP2-Gs vs. EP3-Gi), enabling the development of highly selective ligands (Meng et al., 2025; Huang et al., 2023). This informs a therapeutic paradigm of bidirectional modulation: suppressing pathogenic receptors (EP1, EP2, EP3) while activating protective ones (EP4), tailored to specific disease contexts—a strategy that is exemplified in Table 1, where representative agents and their mechanisms are organized by disease category, allowing readers to compare the divergent use of the same EP receptor (e.g., EP4 agonism in cardioprotection vs. EP4 antagonism in oncology).

**TABLE 1:** Therapeutic targeting and strategies for the PGE_2_ signaling pathway.

Disease category	Target/Strategy	Representative agent(s)	Key mechanism and rationale	References(s)
Neurodegenerative diseases	Alzheimer’s disease (AD)	EP2 antagonist + EP4 agonist	EP2 antagonist: Reduces EP2-driven neuroinflammation. EP4 agonist: Exerts anti-inflammatory and pro-phagocytic effects	Woodling et al. (2014), Wang J. X. et al. (2025), Nango et al. (2023)
​	Parkinson’s disease (PD)	EP1 antagonist + EP4 agonist	EP1 antagonist: Blocks Ca^2+^-mediated neurotoxicity. EP4 agonist: Provides neuroprotection	Woodling et al. (2014), Kawano et al. (2006)
​	Amyotrophic lateral sclerosis (ALS)	EP2 antagonist	Inhibits glial EP2 signaling driving neuroinflammation and motor neuron apoptosis	Nango et al. (2023)
Cardiovascular	Myocardial protection	EP4 agonist	Attenuates ischemia-reperfusion injury via anti-inflammatory effects	Kanno et al. (2023)
​	Atherosclerosis	Bone Marrow EP4 activation	Promotes anti-inflammatory plaque microenvironment	Tang et al. (2011)
​	Cerebrovascular protection	EP4 agonist	Mediates protective vasodilation post-ischemia	Chen et al. (2025)
​	Heart failure	EP3 antagonist	Improves myocardial contractility	Bosma et al. (2022)
Cancer	Tumor immune evasion	EP4 antagonist	Blocks PGE_2_/EP4 axis, reducing VEGF and MDSC-mediated immunosuppression	Thumkeo et al. (2022)
​	​	Dual EP2/EP4 antagonist	Synergistically reprograms immunosuppressive TME.	Punyawatthananukool et al. (2024)
Ageing-related disorders	Muscle regeneration	15-PGDH inhibitor/Exogenous PGE_2_	Restores PGE_2_ to activate CREB pathway in muscle stem cells	Palla et al. (2021), Bakooshli et al. (2023)
​	Immunosenescence	Macrophage EP2 inhibition	Reverses age-related T-cell dysfunction	Punyawatthananukool et al. (2024)
Chronic inflammatory and autoimmune	Rheumatoid arthritis	EP4 antagonist	Inhibits EP4-driven pathogenic Th17 differentiation	Caselli et al. (2018)
​	Multiple sclerosis	EP4 antagonist/Dual COX/5-LO inhibitor	Suppresses Th1/Th17 differentiation; modulates macrophage/microglia	Kong et al. (2016)
​	Diabetic nephropathy	EP1 antagonist	Reduces proteinuria and renal injury via EP1 blockade	Du et al. (2024)
​	Osteoarthritis	EP4 antagonist	Inhibits EP4-mediated osteoclast activation in bone	Jiang et al. (2022)
​	Diabetes/Obesity	EP3 antagonist	Improves glucose homeostasis and reduces adipose inflammation	Shang et al. (2025), Chan et al. (2016)

In neurodegenerative diseases, the detrimental role of EP2 in driving neuroinflammation is evident across AD, PD, and ALS, making its antagonism (e.g., with PF-04418948) a central strategy (Wang J. et al., 2025; Nango et al., 2023). Conversely, EP4 agonism (e.g., with ONO-AE1-329) exerts neuroprotection by suppressing inflammatory responses and enhancing microglial function (Woodling et al., 2014). In PD specifically, EP1 antagonism (e.g., with ONO-8713) also shows promise by mitigating Ca^2+^-mediated neurotoxicity forming a combined strategy of EP1 blockade and EP4 activation (Kawano et al., 2006). Within the cardiovascular and cerebrovascular systems, EP4 agonism is predominantly protective. It attenuates myocardial ischemia-reperfusion injury, promotes atherosclerotic plaque stability (e.g., via bone marrow EP4 activation), and mediates crucial protective vasodilation in the cerebral microvasculature following stroke (Kanno et al., 2023; Chen et al., 2025; Tang et al., 2011). In contrast, for heart failure, EP3 antagonism emerges as a strategy to improve myocardial contractility (Bosma et al., 2022). In oncology, the role of EP4 is starkly reversed. The PGE_2_/EP4 axis is a master regulator of tumor immune evasion, making EP4 antagonists (e.g., L-161,982) promising investigational agents that block this axis, reducing VEGF and MDSC infiltration (Thumkeo et al., 2022). Dual EP2/EP4 antagonism represents a more comprehensive strategy, synergistically reprogramming the immunosuppressive tumor microenvironment to enhance cytotoxic T-cell activity (Punyawatthananukool et al., 2024). For chronic inflammatory and autoimmune diseases, receptor targeting is equally nuanced. EP4 antagonism (e.g., with CR6086 in rheumatoid arthritis or E7046 in multiple sclerosis models) attenuates disease by inhibiting the differentiation of pathogenic Th17 cells (Caselli et al., 2018; Kong et al., 2016). In diabetic nephropathy, EP1 antagonists (e.g., SC-51322) show therapeutic potential by reducing proteinuria and renal injury (Du et al., 2024). In osteoarthritis, EP4 antagonists (e.g., HL-43) alleviate joint destruction by inhibiting osteoclast activation (Jiang et al., 2022). Furthermore, in metabolic disorders, EP3 antagonism can improve glucose homeostasis and adipose tissue inflammation (Shang et al., 2025; Chan et al., 2016). Beyond immune modulation, in ageing, strategies include inhibiting the PGE_2_-degrading enzyme 15-PGDH (e.g., with SW033291) or supplementing exogenous PGE_2_ to promote muscle regeneration, and inhibiting macrophage EP2 signaling to reverse age-related T-cell dysfunction (Palla et al., 2021; Bakooshli et al., 2023; Punyawatthananukool et al., 2024). Collectively, the EP receptor-targeting strategies outlined above exemplify a shift from broad anti-inflammatory therapy to precision medicine. By exploiting the functional duality of the PGE_2_ system—suppressing pathogenic signaling while enhancing protective pathways—these approaches offer context-dependent interventions with the potential to improve therapeutic outcomes across a diverse range of diseases.

### 4.3 Targeting key downstream pathways of PGE_2_–EP signaling

Targeting the specific intracellular signaling cascades activated by PGE_2_-bound EP receptors represents a sophisticated strategy to achieve therapeutic precision while minimizing the systemic drawbacks of COX inhibition. This approach focuses on modulating core downstream pathways whose activity is contextually shaped by upstream receptor engagement.

The cAMP/PKA pathway, primarily activated by EP2/EP4, exemplifies this context-dependence. While generally anti-inflammatory, in the tumor microenvironment (TME), it paradoxically drives immunosuppression and immune evasion (Tang et al., 2025). The PI3K/Akt/mTOR and MAPK/ERK pathways are major effectors of EP4-mediated signaling, promoting cell survival, proliferation, and migration in cancer and inflammatory diseases (Xu et al., 2022; Vo et al., 2013). This overlap suggests rational combination therapies, such as EP4 antagonists with PI3K or MEK inhibitors. A critical target is the β-catenin/Wnt pathway, which is stabilized by the PGE_2_–EP2/EP4–cAMP/PKA axis to promote cancer stemness (Fujino et al., 2015). Targeting the upstream PGE_2_ axis offers a strategic alternative to direct Wnt inhibition. Finally, NF-κB signaling is dually regulated—suppressed via EP2/4–cAMP–PKA or activated via EP1–Ca^2+^—highlighting the net outcome’s dependence on the specific receptor profile. In summary, a “second order” precision strategy involves targeting the key downstream pathways (cAMP/PKA, PI3K/Akt/mTOR, MAPK/ERK, Wnt/β-catenin) that are selectively activated by pathogenic PGE_2_ signaling in specific diseases. This approach enables highly contextual intervention and provides a rationale for novel drug combinations to overcome therapeutic resistance.

## 5 Conclusion and prospectives

This review delineates the complex regulatory landscape of prostaglandin E_2_ (PGE_2_) signaling, wherein the tissue-specific activation of its four receptor subtypes (EP1-EP4) underpins their functional duality in inflammation, cancer, and neurodegeneration. To transcend the limitations of conventional NSAIDs, we propose a precision therapeutic framework encompassing three strategic tiers: modulating PGE_2_ levels via upstream enzymes such as mPGES-1 and 15-PGDH; enabling context-dependent receptor interventions exemplified by the opposing use of EP4 agonists in cardiovascular disease versus antagonists in cancer; and implementing synergistic downstream pathway blockade, for instance through combined EP4 and PI3K/Akt inhibition. Significant translational gaps remain, however. The structural mechanisms behind receptor functional heterogeneity, such as EP4’s opposing roles in different diseases, are still unresolved. Our understanding of the dynamic PGE_2_ microenvironment within pathological niches like the tumor immune landscape is insufficient, and the translational pipeline is hindered by limited ligand selectivity and inadequate preclinical models that fail to fully recapitulate human pathophysiology. Future progress hinges on converging advanced techniques with targeted drug design. Achieving deeper mechanistic insight through structural biology and novel biosensors must be coupled with the innovation of smarter therapeutics, such as tissue-targeted delivery systems and allosteric modulators that fine-tune receptor signaling. Ultimately, closing the translation gap will require leveraging humanized disease models and biomarker-stratified clinical trials. Integrating multi-omics approaches to map the PGE_2_ interactome will be crucial, driving an iterative “mechanism to therapy” cycle that enables truly dynamic and precise targeting of this pathway across a spectrum of diseases. To crystallize what this review has established and where the field must go: the most promising near-term directions include biased EP4 ligands selectively engaging anti-inflammatory Gs/cAMP signaling while avoiding PI3K/β-arrestin-mediated pro-tumorigenic pathways; combination strategies pairing EP receptor-targeted agents with immune checkpoint inhibitors, which have compelling preclinical rationale and are beginning to enter trial design; and 15-PGDH inhibition for muscle regeneration in aging, a mechanistically novel application with strong proof-of-concept data. The key barriers are (Badimon et al., 2021): mechanistic—the structural basis for EP4’s opposing roles across disease contexts remains unresolved, limiting rational patient selection (Kanaoka and Austen, 2019); translational—validated biomarkers for PGE_2_ pathway activity in human tissues are lacking, making on-target engagement difficult to confirm in clinical trials; and (Zhang et al., 2023) clinical—the most robust human evidence still derives from COX inhibitor and NSAID trials; EP receptor-selective agents remain at early Phase I/II stages without definitive efficacy data, and this gap between strong preclinical rationale and limited clinical validation must be explicitly acknowledged as the field moves forward. Three frontier questions define the next decade: What molecular determinants govern EP receptor subtype dominance in a given pathological niche? How does signaling kinetics interact with disease chronicity to shift PGE_2_ outputs from resolution to pathology? And can pathway-biased EP4 ligands be rationally designed to separate therapeutic from adverse signaling?
